# Supplementary material for: Increased ultra-rare variant load in an isolated Scottish population impacts exonic and regulatory regions
Source: PLoS Genet. 2019 Nov 25;15(11):e1008480. doi: 10.1371/journal.pgen.1008480 (PMC6901239; doi:10.1371/journal.pgen.1008480)
Supplement: S5 Fig — Circle dots represent the ratio of the median number of variants in a VIKING individual to the median number of variants in an LBC individual; whiskers are 95% CI based in 10k randomly selected LBC subsets (n = 269). Significance: at least 95% of the 10k subsets have p-value ≤ 2x10^-4^ (Bonferroni corrected) and no overlap between the 95% CI for the LBC median and the VIKING median value. Red vertical lines represent the median genome-wide enrichment for ultra-rare INDELs and its 95% CI. No significant difference was observed for any of the cell types in the insulator regions (not plotted). (PDF) [file pgen.1008480.s005.pdf]

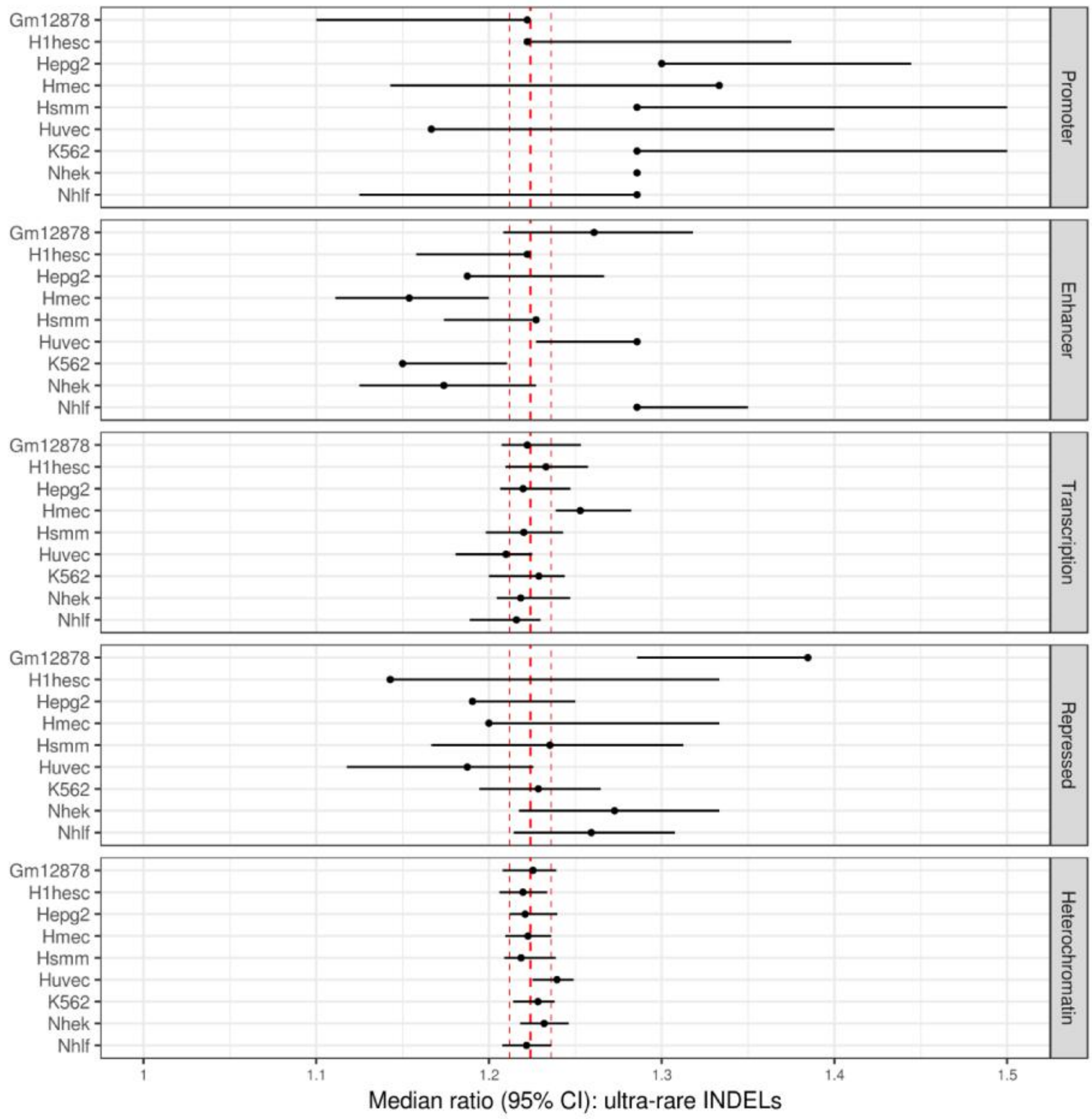

S5 Fig. Significant differences in variant load in regulatory regions for ultra-rare INDELs in 9 cell types.
