# Supplementary material for: Increased ultra-rare variant load in an isolated Scottish population impacts exonic and regulatory regions
Source: PLoS Genet. 2019 Nov 25;15(11):e1008480. doi: 10.1371/journal.pgen.1008480 (PMC6901239; doi:10.1371/journal.pgen.1008480)
Supplement: S11 Fig — The red points depict data from the 34 unrelated (pi_hat = 0) VIK individuals. Black boxplots represent the data from 10 control subsets of 34 VIK individuals randomly selected from the remaining 466 VIK individuals (without replacement within subsets, with replacement across subsets). The upper and lower "hinges" correspond to the first and third quartiles (the 25th and 75th percentiles); the upper whisker extends from the hinge to the highest value that is within 1.5 * IQR of the hinge, where IQR is the inter-quartile range, or distance between the first and third quartiles; the lower whisker extends from the hinge to the lowest value within 1.5 * IQR of the hinge. Top panel: sites split to those with minor AC from 1 to 9 and 10+, lower panel: zoom in onto rarer sites with minor AC = 1, 2, 3, 4 and 5. (PDF) [file pgen.1008480.s011.pdf]

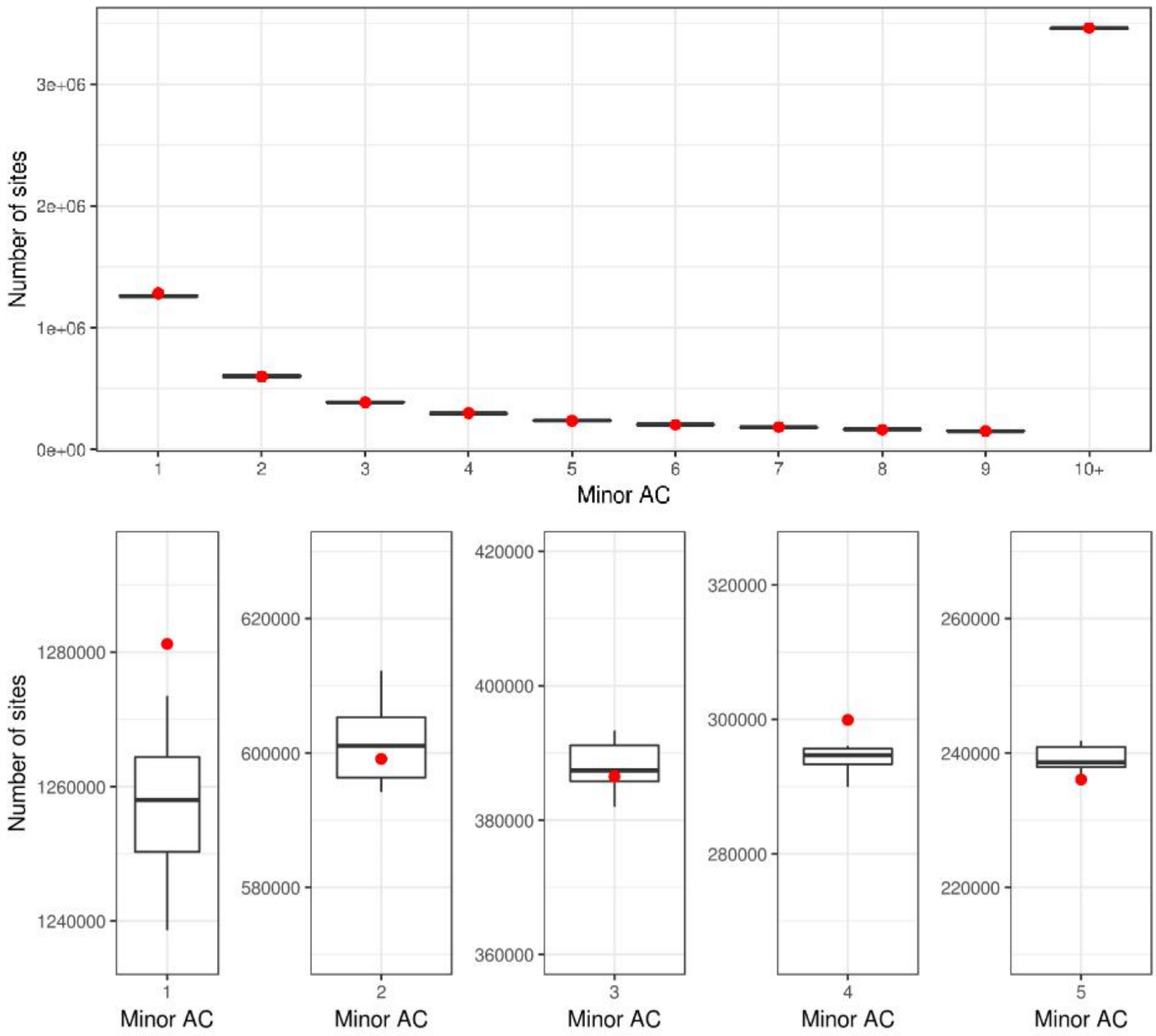

S11 Fig. KEEP_IF_ANY_UNFILTERED does not introduce a bias towards rarer variants in more related populations.
