# Supplementary material for: Increased ultra-rare variant load in an isolated Scottish population impacts exonic and regulatory regions
Source: PLoS Genet. 2019 Nov 25;15(11):e1008480. doi: 10.1371/journal.pgen.1008480 (PMC6901239; doi:10.1371/journal.pgen.1008480)
Supplement: S1 Table — The variants are stratified by their presence in the full gnomAD genomes dataset (n = 15,496) and their prevalence in gnomAD Non-Finnish Europeans (NFE) population (n = 7,509). (PDF) [file pgen.1008480.s014.pdf]

S1 Table. Average number of high-quality variant alleles found per unrelated individual in the VIKING and LBC cohorts.

gnomADg MAF	VIKING (n=269)		LBC (n=1156)	
	SNP (s.d.)	INDEL (s.d.)	SNP (s.d.)	INDEL (s.d.)
**Total**	3,528,153	356,552	3,524,508	354,424
**very common**: $MAF_{NFE} > 10\%$	3,287,505 (8,697)	331,347 (1,041)	3,283,725 (8,867)	329,440 (1,322)
**common**: $5\% < MAF_{NFE} \leq 10\%$	115,419 (2,166)	11,954 (249)	115,316 (2,217)	11,805 (264)
**rare**: $1\% < MAF_{NFE} \leq 5\%$	86,203 (1,619)	8,730 (215)	86,513 (1,732)	8,662 (214)
**very rare**: $MAF_{NFE} \leq 1\%$	33,857 (792)	4,017 (106)	34,481 (1,873)	4,104 (206)
**ultra-rare**: not in gnomADg	5,169 (164)	504 (28)	4,472 (410)	413 (40)
