# Supplementary material for: Increased ultra-rare variant load in an isolated Scottish population impacts exonic and regulatory regions
Source: PLoS Genet. 2019 Nov 25;15(11):e1008480. doi: 10.1371/journal.pgen.1008480 (PMC6901239; doi:10.1371/journal.pgen.1008480)
Supplement: S3 Table — Very common: variants with MAF > 10% in Non-Finnish Europeans (NFE, gnomADg, n = 7,509); common: 5% < MAFNFE ≤ 10%; rare: 1% < MAFNFE ≤ 5%; very rare: MAFNFE ≤ 1%; ultra-rare: not observed in any gnomADg individual (n = 15,496). Median number and 95% CI of LBC alleles (forth column) for each frequency class is computed based on 10,000 random subsets (n = 269, matching VIKING size); last two columns represent the median p-value (and 95% CI) and the number of tests with p-value smaller than the Bonferroni corrected threshold. To annotate the number of variants in a frequency class as significantly different (shown in bold), we required at least 95% of the 10,000 subsets to have p-value ≤ 8x10^-4^ (Bonferroni corrected) and no overlap between the 95% CI for the LBC and VIKING median values. Region annotation (5’UTR, Exon, Intron, 3’UTR, ncRNA) is based on Ensembl data (GRCh37.p13, Ensembl Genes 92) for the mappable sections of the 22 autosomal chromosomes; the remaining 1.1Gb of the mappable regions in the reference human genome is annotated as “non-coding”. (PDF) [file pgen.1008480.s016.pdf]

S3 Table. VIKING vs LBC: INDEL load comparison in coding and coding related regions (alleles per individual per 1Mb).

Region	gnomAD Frequency Class	VIKING median	LBC 10k subsets median & 95%CI	VIKING/LBC ratio median & 95%CI	Wilcoxon rank sum test	
					p : median & 95% CI	number of tests with $p \leq 8\times10^{-4}$
5'UTR	**very common**	**102.08**	**100.90 [100.57, 101.22]**	**1.012 [1.009, 1.015]**	**1.2×10^{-8} [7.4×10^{-12}, 4.9×10^{-6}]**	**10000**
	common	3.98	3.77 [3.66, 3.88]	1.057 [1.028, 1.088]	2.5×10^{-3} [2.6×10^{-5}, 6.7×10^{-2}]	2990
	rare	3.12	3.02 [2.91, 3.12]	1.036 [1.000, 1.074]	1.7×10^{-2} [3.6×10^{-4}, 2.5×10^{-1}]	581
	very rare	1.72	1.72 [1.72, 1.83]	1.000 [0.941, 1.000]	4.8×10^{-1} [5.1×10^{-2}, 9.7×10^{-1}]	0
	ultra-rare	0.22	0.11 [0.11, 0.22]	2.000 [1.000, 2.000]	3.7×10^{-5} [1.5×10^{-7}, 2.7×10^{-3}]	9086
Exon	very common	12.51	12.44 [12.41, 12.54]	1.005 [0.997, 1.008]	3.6×10^{-1} [2.7×10^{-2}, 9.6×10^{-1}]	1
	common	0.60	0.63 [0.60, 0.67]	0.947 [0.900, 1.000]	6.7×10^{-1} [1.5×10^{-1}, 9.8×10^{-1}]	0
	rare	0.57	0.53 [0.53, 0.57]	1.062 [1.000, 1.062]	9.9×10^{-2} [5.1×10^{-3}, 6.1×10^{-1}]	19
	very rare	0.57	0.60 [0.60, 0.63]	0.944 [0.895, 0.944]	5.3×10^{-2} [2.1×10^{-3}, 4.1×10^{-1}]	65
	ultra-rare	0.10	0.10 [0.10, 0.10]	1.000 [1.000, 1.0000]	1.1×10^{-1} [5.3×10^{-3}, 6.7×10^{-1}]	20
Intron	**very common**	**154.31**	**153.41 [153.32, 153.49]**	**1.006 [1.005, 1.006]**	**1.3×10^{-41} [4.2×10^{-47}, 2.4×10^{-36}]**	**10000**
	**common**	**5.64**	**5.56 [5.54, 5.58]**	**1.014 [1.010, 1.018]**	**2.3×10^{-8} [1.2×10^{-11}, 1.3×10^{-5}]**	**9999**
	rare	4.23	4.19 [4.17, 4.21]	1.009 [1.005, 1.013]	4.4×10^{-4} [2.4×10^{-6}, 2.2×10^{-2}]	6058
	**very rare**	**1.86**	**1.90 [1.89, 1.91]**	**0.978 [0.974, 0.983]**	**6.0×10^{-10} [1.9×10^{-13}, 5.6×10^{-7}]**	**10000**
	**ultra-rare**	**0.25**	**0.20 [0.20, 0.21]**	**1.228 [1.215, 1.249]**	**1.4×10^{-74} [9.8×10^{-79}, 3.8×10^{-70}]**	**10000**
3'UTR	**very common**	**160.43**	**159.63 [159.41, 159.88]**	**1.005 [1.003, 1.006]**	**3.4×10^{-8} [1.5×10^{-11}, 1.6×10^{-5}]**	**9997**
	common	6.27	6.17 [6.09, 6.20]	1.018 [1.012, 1.03]	1.9×10^{-2} [5.6×10^{-4}, 2.3×10^{-1}]	376
	rare	4.79	4.82 [4.75, 4.90]	0.992 [0.978, 1.008]	6.8×10^{-1} [1.7×10^{-1}, 9.8×10^{-1}]	0
	very rare	2.25	2.39 [2.36, 2.43]	0.939 [0.925, 0.954]	9.8×10^{-5} [3.6×10^{-7}, 6.6×10^{-3}]	8218
	ultra-rare	0.29	0.25 [0.25, 0.25]	1.143 [1.143, 1.143]	1.3×10^{-3} [8.5×10^{-6}, 4.6×10^{-2}]	4065
ncRNA	very common	149.13	148.58 [148.17, 149.00]	1.004 [1.001, 1.006]	1.6×10^{-1} [8.8×10^{-3}, 8.3×10^{-1}]	15
	common	6.04	5.76 [5.63, 5.90]	1.048 [1.023, 1.073]	1.3×10^{-3} [1.2×10^{-5}, 3.6×10^{-2}]	4150
	rare	4.12	3.98 [3.84, 4.12]	1.034 [1.000, 1.071]	5.4×10^{-1} [7.8×10^{-2}, 9.8×10^{-1}]	0
	very rare	2.33	2.33 [2.33, 2.47]	1.000 [0.944, 1.000]	5.5×10^{-1} [7.9×10^{-2}, 9.8×10^{-1}]	0
	ultra-rare	0.14	0.14 [0.14, 0.14]	1.000 [1.000, 1.000]	1.6×10^{-2} [4.2×10^{-4}, 2.0×10^{-1}]	496
non-coding	**very common**	**171.56**	**170.64 [170.54, 170.74]**	**1.005 [1.005, 1.006]**	**3.8×10^{-38} [2.8×10^{-43}, 4.4×10^{-33}]**	**10000**
	common	6.11	6.06 [6.03, 6.08]	1.009 [1.005, 1.013]	3.4×10^{-5} [1.2×10^{-7}, 2.8×10^{-3}]	9113
	rare	4.37	4.35 [4.33, 4.36]	1.006 [1.001, 1.010]	2.4×10^{-2} [5.5×10^{-4}, 3.0×10^{-1}]	366
	**very rare**	**2.06**	**2.10 [2.09, 2.11]**	**0.980 [0.977, 0.984]**	**1.7×10^{-9} [1.7×10^{-12}, 7.5×10^{-7}]**	**10000**
	**ultra-rare**	**0.25**	**0.20 [0.20, 0.20]**	**1.214 [1.203, 1.230]**	**6.5×10^{-71} [1.9×10^{-75}, 4.3×10^{-66}]**	**10000**
