# Supplementary material for: Increased ultra-rare variant load in an isolated Scottish population impacts exonic and regulatory regions
Source: PLoS Genet. 2019 Nov 25;15(11):e1008480. doi: 10.1371/journal.pgen.1008480 (PMC6901239; doi:10.1371/journal.pgen.1008480)
Supplement: S4 Table — To annotate the number of variants in a state/cell type class as significantly different, we required at least 95% of the 10,000 subsets to have p-value ≤ 2x10^-4^ (Bonferroni corrected) and no overlap between the 95% CI for the LBC and VIKING median values; VIKING is enriched for ultra-rare SNPs in all considered states/cell types. (PDF) [file pgen.1008480.s017.pdf]

S4 Table. VIKING vs LBC: ultra-rare SNP load comparison in different chromatin states (alleles per individual per 1Mb).

State	Cell Type	VIKING median	LBC 10k subsets median & 95%CI	VIKING/LBC ratio median & 95%CI	Wilcoxon rank sum test: p : median & 95% CI	Wilcoxon rank sum test: number of tests with $p \leq 2\times10^{-4}$
Promoter	Gm12878	2.379	2.005 [1.961, 2.049]	1.187 [1.161, 1.213]	7.1×10^{-39} [1.3×10^{-44}, 3.0×10^{-33}]	10000
	H1hesc	2.391	2.000 [1.978, 2.043]	1.196 [1.170, 1.209]	4.8×10^{-50} [2.3×10^{-55}, 9.8×10^{-45}]	10000
	Hepg2	2.265	1.928 [1.909, 1.965]	1.175 [1.152, 1.186]	4.6×10^{-44} [2.2×10^{-49}, 8.1×10^{-39}]	10000
	Hmec	2.378	1.987 [1.957, 2.017]	1.197 [1.179, 1.215]	2.0×10^{-39} [6.4×10^{-45}, 4.9×10^{-34}]	10000
	Hsmm	2.372	1.986 [1.958, 2.041]	1.194 [1.162, 1.211]	2.2×10^{-43} [9.7×10^{-49}, 4.4×10^{-38}]	10000
	Huvec	2.378	2.009 [1.942, 2.043]	1.183 [1.164, 1.224]	1.4×10^{-41} [9.0×10^{-47}, 2.0×10^{-36}]	10000
	K562	2.208	1.897 [1.869, 1.954]	1.164 [1.130, 1.182]	3.0×10^{-32} [2.2×10^{-37}, 2.3×10^{-27}]	10000
	Nhek	2.453	2.040 [2.012, 2.095]	1.203 [1.171, 1.219]	5.7×10^{-40} [1.1×10^{-45}, 2.1×10^{-34}]	10000
	Nhlf	2.352	2.001 [1.974, 2.028]	1.176 [1.160, 1.192]	5.8×10^{-45} [3.0×10^{-50}, 1.1×10^{-39}]	10000
	Union	2.302	1.951 [1.923, 1.970]	1.180 [1.168, 1.197]	5.9×10^{-56} [2.0×10^{-61}, 1.6×10^{-50}]	10000
Enhancer	Gm12878	2.091	1.760 [1.745, 1.790]	1.188 [1.168, 1.198]	2.2×10^{-53} [2.0×10^{-59}, 2.8×10^{-47}]	10000
	H1hesc	2.189	1.889 [1.862, 1.907]	1.159 [1.148, 1.176]	7.2×10^{-54} [4.4×10^{-59}, 1.3×10^{-48}]	10000
	Hepg2	1.964	1.685 [1.666, 1.714]	1.166 [1.146, 1.179]	1.2×10^{-53} [6.6×10^{-59}, 2.3×10^{-48}]	10000
	Hmec	2.085	1.806 [1.787, 1.818]	1.155 [1.147, 1.167]	9.1×10^{-65} [1.4×10^{-69}, 9.9×10^{-60}]	10000
	Hsmm	2.153	1.853 [1.839, 1.875]	1.161 [1.148, 1.171]	1.7×10^{-61} [4.2×10^{-66}, 9.8×10^{-57}]	10000
	Huvec	2.117	1.801 [1.777, 1.825]	1.175 [1.160, 1.191]	1.0×10^{-64} [2.5×10^{-69}, 6.2×10^{-60}]	10000
	K562	1.896	1.621 [1.598, 1.636]	1.170 [1.159, 1.186]	1.3×10^{-64} [2.3×10^{-69}, 1.1×10^{-59}]	10000
	Nhek	2.069	1.800 [1.779, 1.814]	1.150 [1.141, 1.163]	1.4×10^{-62} [1.0×10^{-67}, 2.5×10^{-57}]	10000
	Nhlf	2.139	1.854 [1.831, 1.878]	1.153 [1.139, 1.168]	4.7×10^{-53} [1.3×10^{-58}, 1.8×10^{-47}]	10000
	Union	2.056	1.773 [1.761, 1.788]	1.160 [1.150, 1.168]	2.8×10^{-78} [1.1×10^{-82}, 1.7×10^{-73}]	10000
Insulator	Gm12878	2.136	1.869 [1.802, 1.935]	1.143 [1.103, 1.185]	9.4×10^{-12} [1.9×10^{-15}, 1.3×10^{-8}]	10000
	H1hesc	2.106	1.922 [1.877, 1.968]	1.095 [1.070, 1.122]	2.8×10^{-16} [9.8×10^{-21}, 1.9×10^{-12}]	10000
	Hepg2	2.150	1.978 [1.892, 1.978]	1.087 [1.087, 1.136]	1.8×10^{-6} [1.3×10^{-9}, 2.8×10^{-4}]	9661
	Hmec	2.072	1.913 [1.833, 1.913]	1.083 [1.083, 1.130]	7.6×10^{-10} [1.9×10^{-13}, 7.7×10^{-7}]	10000
	Hsmm	2.252	1.931 [1.931, 1.995]	1.167 [1.129, 1.167]	3.9×10^{-12} [5.8×10^{-16}, 5.8×10^{-9}]	10000
	Huvec	2.191	1.859 [1.792, 1.925]	1.179 [1.138, 1.222]	4.6×10^{-16} [1.7×10^{-20}, 4.3×10^{-12}]	10000
	K562	2.138	1.877 [1.825, 1.929]	1.139 [1.108, 1.171]	4.5×10^{-20} [5.3×10^{-25}, 9.8×10^{-16}]	10000
	Nhek	2.189	1.876 [1.824, 1.928]	1.167 [1.135, 1.200]	7.6×10^{-20} [1.5×10^{-24}, 1.4×10^{-15}]	10000
	Nhlf	2.097	1.830 [1.792, 1.869]	1.146 [1.122, 1.170]	1.4×10^{-26} [1.2×10^{-31}, 9.0×10^{-22}]	10000
	Union	2.165	1.895 [1.861, 1.911]	1.143 [1.133, 1.164]	1.4×10^{-38} [6.2×10^{-44}, 1.4×10^{-33}]	10000
Transcription	Gm12878	1.895	1.650 [1.637, 1.665]	1.149 [1.138, 1.158]	1.2×10^{-66} [7.9×10^{-72}, 3.2×10^{-61}]	10000
	H1hesc	1.927	1.670 [1.655, 1.682]	1.154 [1.146, 1.164]	5.9×10^{-76} [1.6×10^{-80}, 7.5×10^{-71}]	10000
	Hepg2	1.858	1.615 [1.602, 1.626]	1.151 [1.143, 1.160]	1.2×10^{-73} [2.5×10^{-78}, 1.4×10^{-68}]	10000
	Hmec	1.893	1.646 [1.633, 1.658]	1.150 [1.142, 1.159]	3.4×10^{-73} [5.4×10^{-78}, 4.0×10^{-68}]	10000
	Hsmm	1.887	1.637 [1.624, 1.649]	1.153 [1.144, 1.162]	5.6×10^{-75} [1.4×10^{-79}, 5.5×10^{-70}]	10000
	Huvec	1.873	1.633 [1.618, 1.650]	1.147 [1.135, 1.157]	3.9×10^{-70} [5.7×10^{-75}, 5.7×10^{-65}]	10000
	K562	1.816	1.574 [1.564, 1.588]	1.154 [1.144, 1.161]	2.0×10^{-74} [4.0×10^{-79}, 3.0×10^{-69}]	10000
	Nhek	1.864	1.613 [1.604, 1.627]	1.156 [1.146, 1.162]	1.3×10^{-73} [3.4×10^{-78}, 1.4×10^{-68}]	10000
	Nhlf	1.878	1.634 [1.620, 1.649]	1.149 [1.139, 1.159]	1.2×10^{-73} [2.8×10^{-78}, 1.3×10^{-68}]	10000
	Union	1.911	1.655 [1.643, 1.668]	1.155 [1.146, 1.163]	9.0×10^{-81} [6.6×10^{-85}, 4.0×10^{-76}]	10000
Repressed	Gm12878	2.062	1.803 [1.769, 1.825]	1.144 [1.130, 1.166]	1.3×10^{-47} [3.5×10^{-53}, 3.8×10^{-42}]	10000
	H1hesc	2.076	1.810 [1.757, 1.837]	1.147 [1.130, 1.182]	3.1×10^{-25} [6.5×10^{-31}, 3.8×10^{-20}]	10000
	Hepg2	2.202	1.908 [1.884, 1.925]	1.154 [1.144, 1.169]	7.8×10^{-50} [1.6×10^{-55}, 3.0×10^{-44}]	10000
	Hmec	2.223	1.927 [1.894, 1.960]	1.154 [1.134, 1.174]	2.3×10^{-35} [3.8×10^{-41}, 7.5×10^{-30}]	10000
	Hsmm	2.075	1.795 [1.778, 1.821]	1.156 [1.140, 1.167]	1.8×10^{-47} [1.2×10^{-52}, 3.4×10^{-42}]	10000
	Huvec	2.070	1.774 [1.748, 1.799]	1.167 [1.150, 1.184]	3.6×10^{-51} [5.4×10^{-57}, 2.0×10^{-45}]	10000
	K562	2.088	1.818 [1.800, 1.837]	1.148 [1.137, 1.160]	5.3×10^{-64} [2.6×10^{-69}, 1.4×10^{-58}]	10000
	Nhek	2.206	1.928 [1.901, 1.949]	1.144 [1.132, 1.161]	2.7×10^{-50} [4.8×10^{-56}, 8.0×10^{-45}]	10000
	Nhlf	2.007	1.756 [1.729, 1.772]	1.143 [1.133, 1.160]	4.3×10^{-56} [7.8×10^{-62}, 3.1×10^{-50}]	10000
	Union	2.074	1.787 [1.773, 1.802]	1.161 [1.151, 1.170]	1.8×10^{-76} [6.4×10^{-81}, 1.0×10^{-71}]	10000
Heterochromatin	Gm12878	1.896	1.631 [1.620, 1.641]	1.162 [1.155, 1.171]	3.1×10^{-82} [3.1×10^{-86}, 1.4×10^{-77}]	10000
	H1hesc	1.890	1.623 [1.611, 1.635]	1.164 [1.156, 1.173]	1.2×10^{-81} [1.3×10^{-85}, 5.1×10^{-77}]	10000
	Hepg2	1.908	1.640 [1.627, 1.654]	1.163 [1.154, 1.172]	8.1×10^{-82} [8.6×10^{-86}, 3.8×10^{-77}]	10000
	Hmec	1.898	1.631 [1.619, 1.642]	1.163 [1.156, 1.172]	5.9×10^{-82} [5.4×10^{-86}, 3.2×10^{-77}]	10000
	Hsmm	1.895	1.627 [1.615, 1.638]	1.165 [1.157, 1.174]	1.1×10^{-81} [1.2×10^{-85}, 5.9×10^{-77}]	10000
	Huvec	1.892	1.629 [1.618, 1.641]	1.162 [1.153, 1.169]	5.4×10^{-82} [5.2×10^{-86}, 2.5×10^{-77}]	10000
	K562	1.920	1.652 [1.640, 1.664]	1.162 [1.154, 1.171]	4.3×10^{-81} [3.1×10^{-85}, 2.4×10^{-76}]	10000
	Nhek	1.893	1.626 [1.610, 1.637]	1.164 [1.156, 1.176]	5.7×10^{-82} [5.8×10^{-86}, 2.5×10^{-77}]	10000
	Nhlf	1.897	1.627 [1.617, 1.639]	1.166 [1.157, 1.173]	5.6×10^{-82} [5.0×10^{-86}, 3.4×10^{-77}]	10000
	Union	1.918	1.650 [1.638, 1.662]	1.163 [1.154, 1.171]	3.8×10^{-82} [3.5×10^{-86}, 1.6×10^{-77}]	10000
