# Supplementary material for: Increased ultra-rare variant load in an isolated Scottish population impacts exonic and regulatory regions
Source: PLoS Genet. 2019 Nov 25;15(11):e1008480. doi: 10.1371/journal.pgen.1008480 (PMC6901239; doi:10.1371/journal.pgen.1008480)
Supplement: S5 Table — To annotate the number of variants in a state/cell type class as significantly different, we required at least 95% of the 10,000 subsets to have p-value ≤ 2x10^-4^ (Bonferroni corrected) and no overlap between the 95% CI for the LBC and VIKING median values; similar to ultra-rare SNPs, VIKING is enriched for ultra-rare INDELs in almost all considered states/cell types, except the Insulator chromatin state (shown in grey). (PDF) [file pgen.1008480.s018.pdf]

S5 Table. VIKING vs LBC: ultra-rare INDEL load comparison in different chromatin states (alleles per individual per 1Mb).

State	Cell Type	VIKING median	LBC 10k subsets median & 95%CI	VIKING/LBC ratio median & 95%CI	Wilcoxon rank sum test	
					p : median & 95% CI	number of tests with $p \leq 2x10^{-4}$
Promoter	Gm12878	0.242	0.198 [0.198, 0.220]	1.222 [1.100, 1.222]	$3.0x10^{-10}$ [$1.2x10^{-13}$, $2.9x10^{-7}$]	9999
	H1hesc	0.239	0.196 [0.174, 0.196]	1.222 [1.222, 1.375]	$7.3x10^{-10}$ [$3.2x10^{-13}$, $5.8x10^{-7}$]	10000
	Hepg2	0.243	0.187 [0.168, 0.187]	1.300 [1.300, 1.444]	$3.6x10^{-20}$ [$2.9x10^{-24}$, $2.7x10^{-16}$]	10000
	Hmec	0.241	0.181 [0.181, 0.211]	1.333 [1.143, 1.333]	$1.9x10^{-10}$ [$1.4x10^{-13}$, $1.0x10^{-7}$]	10000
	Hsmm	0.248	0.193 [0.165, 0.193]	1.286 [1.286, 1.500]	$3.8x10^{-15}$ [$8.0x10^{-19}$, $8.1x10^{-12}$]	10000
	Huvec	0.234	0.201 [0.167, 0.201]	1.167 [1.167, 1.400]	$2.1x10^{-9}$ [$1.7x10^{-12}$, $1.0x10^{-6}$]	10000
	K562	0.255	0.198 [0.170, 0.198]	1.286 [1.286, 1.500]	$1.1x10^{-14}$ [$1.8x10^{-18}$, $3.7x10^{-11}$]	10000
	Nhek	0.248	0.193 [0.193, 0.193]	1.286 [1.286, 1.286]	$1.0x10^{-10}$ [$3.9x10^{-14}$, $9.1x10^{-8}$]	10000
	Nhlf	0.243	0.189 [0.189, 0.216]	1.286 [1.125, 1.286]	$1.4x10^{-8}$ [$1.4x10^{-11}$, $5.7x10^{-6}$]	9993
Enhancer	Gm12878	0.218	0.173 [0.165, 0.181]	1.261 [1.208, 1.318]	$3.3x10^{-19}$ [$7.4x10^{-24}$, $5.7x10^{-15}$]	10000
	H1hesc	0.200	0.163 [0.163, 0.173]	1.222 [1.158, 1.222]	$1.3x10^{-17}$ [$5.9x10^{-22}$, $8.9x10^{-14}$]	10000
	Hepg2	0.183	0.154 [0.144, 0.154]	1.187 [1.187, 1.267]	$3.7x10^{-16}$ [$1.8x10^{-20}$, $2.8x10^{-12}$]	10000
	Hmec	0.186	0.161 [0.155, 0.168]	1.154 [1.111, 1.200]	$3.5x10^{-20}$ [$6.9x10^{-25}$, $5.7x10^{-16}$]	10000
	Hsmm	0.197	0.161 [0.161, 0.168]	1.227 [1.174, 1.227]	$1.2x10^{-25}$ [$3.7x10^{-30}$, $2.4x10^{-21}$]	10000
	Huvec	0.213	0.166 [0.166, 0.174]	1.286 [1.227, 1.286]	$2.4x10^{-30}$ [$2.7x10^{-35}$, $1.4x10^{-25}$]	10000
	K562	0.171	0.149 [0.141, 0.149]	1.150 [1.150, 1.211]	$4.6x10^{-19}$ [$1.6x10^{-23}$, $3.5x10^{-15}$]	10000
	Nhek	0.191	0.163 [0.156, 0.170]	1.174 [1.125, 1.227]	$8.6x10^{-23}$ [$1.3x10^{-27}$, $1.6x10^{-18}$]	10000
	Nhlf	0.213	0.166 [0.158, 0.166]	1.286 [1.286, 1.350]	$9.2x10^{-30}$ [$2.8x10^{-34}$, $1.7x10^{-25}$]	10000
Insulator	Gm12878	0.200	0.133 [0.133, 0.200]	1.500 [1.000, 1.500]	$4.2x10^{-3}$ [$5.3x10^{-5}$, $9.3x10^{-2}$]	782
	H1hesc	0.183	0.183 [0.137, 0.183]	1.000 [1.000, 1.333]	$2.7x10^{-3}$ [$3.3x10^{-5}$, $6.4x10^{-2}$]	1148
	Hepg2	0.172	0.172 [0.086, 0.172]	1.000 [1.000, 2.000]	$7.4x10^{-2}$ [$3.2x10^{-3}$, $5.4x10^{-1}$]	3
	Hmec	0.159	0.159 [0.159, 0.159]	1.000 [1.000, 1.000]	$5.8x10^{-2}$ [$1.6x10^{-3}$, $4.8x10^{-1}$]	28
	Hsmm	0.193	0.193 [0.193, 0.193]	1.000 [1.000, 1.000]	$2.8x10^{-2}$ [$7.6x10^{-4}$, $3.1x10^{-1}$]	50
	Huvec	0.199	0.133 [0.133, 0.199]	1.500 [1.000, 1.500]	$1.2x10^{-1}$ [$5.4x10^{-3}$, $7.3x10^{-1}$]	1
	K562	0.156	0.156 [0.156, 0.156]	1.000 [1.000, 1.000]	$2.9x10^{-1}$ [$2.7x10^{-2}$, $9.2x10^{-1}$]	0
	Nhek	0.208	0.156 [0.156, 0.156]	1.333 [1.333, 1.333]	$7.6x10^{-5}$ [$2.7x10^{-7}$, $5.8x10^{-3}$]	6425
	Nhlf	0.153	0.153 [0.153, 0.153]	1.000 [1.000, 1.000]	$7.7x10^{-2}$ [$2.9x10^{-3}$, $6.0x10^{-1}$]	9
Transcription	Gm12878	0.211	0.173 [0.169, 0.175]	1.222 [1.207, 1.253]	$5.2x10^{-50}$ [$8.5x10^{-55}$, $3.3x10^{-45}$]	10000
	H1hesc	0.211	0.171 [0.168, 0.174]	1.233 [1.210, 1.257]	$1.2x10^{-64}$ [$7.6x10^{-69}$, $2.6x10^{-60}$]	10000
	Hepg2	0.209	0.171 [0.168, 0.173]	1.220 [1.207, 1.247]	$1.7x10^{-58}$ [$4.3x10^{-63}$, $7.9x10^{-54}$]	10000
	Hmec	0.210	0.168 [0.164, 0.170]	1.253 [1.239, 1.282]	$3.6x10^{-61}$ [$7.7x10^{-66}$, $2.9x10^{-56}$]	10000
	Hsmm	0.205	0.168 [0.165, 0.171]	1.220 [1.198, 1.243]	$3.2x10^{-61}$ [$4.0x10^{-66}$, $2.4x10^{-56}$]	10000
	Huvec	0.206	0.170 [0.168, 0.175]	1.210 [1.181, 1.225]	$2.1x10^{-53}$ [$1.0x10^{-58}$, $4.8x10^{-48}$]	10000
	K562	0.206	0.168 [0.166, 0.172]	1.229 [1.200, 1.244]	$4.2x10^{-56}$ [$3.7x10^{-61}$, $4.0x10^{-51}$]	10000
	Nhek	0.205	0.168 [0.164, 0.170]	1.218 [1.205, 1.247]	$1.2x10^{-57}$ [$9.4x10^{-63}$, $1.9x10^{-52}$]	10000
	Nhlf	0.207	0.171 [0.169, 0.174]	1.216 [1.189, 1.230]	$1.1x10^{-56}$ [$6.4x10^{-62}$, $2.2x10^{-51}$]	10000
Repressed	Gm12878	0.203	0.146 [0.146, 0.158]	1.385 [1.286, 1.385]	$3.0x10^{-25}$ [$1.5x10^{-29}$, $4.9x10^{-21}$]	10000
	H1hesc	0.213	0.186 [0.160, 0.186]	1.143 [1.143, 1.333]	$9.0x10^{-10}$ [$3.8x10^{-13}$, $7.7x10^{-7}$]	10000
	Hepg2	0.204	0.171 [0.163, 0.171]	1.190 [1.190, 1.250]	$1.3x10^{-24}$ [$1.5x10^{-29}$, $5.2x10^{-20}$]	10000
	Hmec	0.198	0.165 [0.148, 0.165]	1.200 [1.200, 1.333]	$1.2x10^{-13}$ [$1.4x10^{-17}$, $3.3x10^{-10}$]	10000
	Hsmm	0.178	0.144 [0.136, 0.152]	1.235 [1.167, 1.312]	$1.1x10^{-20}$ [$3.7x10^{-25}$, $1.9x10^{-16}$]	10000
	Huvec	0.194	0.163 [0.158, 0.173]	1.187 [1.118, 1.226]	$6.5x10^{-17}$ [$2.6x10^{-21}$, $5.8x10^{-13}$]	10000
	K562	0.196	0.160 [0.155, 0.164]	1.229 [1.194, 1.265]	$7.6x10^{-29}$ [$1.2x10^{-33}$, $2.9x10^{-24}$]	10000
	Nhek	0.194	0.153 [0.146, 0.160]	1.273 [1.217, 1.333]	$9.1x10^{-25}$ [$2.0x10^{-29}$, $2.5x10^{-20}$]	10000
	Nhlf	0.181	0.144 [0.139, 0.149]	1.259 [1.214, 1.308]	$2.1x10^{-31}$ [$3.4x10^{-36}$, $8.2x10^{-27}$]	10000
Heterochromatin	Gm12878	0.175	0.143 [0.142, 0.145]	1.225 [1.208, 1.239]	$5.7x10^{-79}$ [$1.9x10^{-82}$, $7.2x10^{-75}$]	10000
	H1hesc	0.174	0.142 [0.141, 0.144]	1.220 [1.206, 1.234]	$2.8x10^{-78}$ [$4.9x10^{-82}$, $7.1x10^{-74}$]	10000
	Hepg2	0.176	0.144 [0.142, 0.145]	1.221 [1.212, 1.240]	$3.9x10^{-77}$ [$4.9x10^{-81}$, $9.3x10^{-73}$]	10000
	Hmec	0.178	0.145 [0.144, 0.147]	1.223 [1.209, 1.236]	$4.6x10^{-78}$ [$9.0x10^{-82}$, $8.3x10^{-74}$]	10000
	Hsmm	0.175	0.144 [0.141, 0.145]	1.219 [1.209, 1.239]	$2.6x10^{-76}$ [$2.2x10^{-80}$, $1.1x10^{-71}$]	10000
	Huvec	0.176	0.142 [0.141, 0.143]	1.239 [1.225, 1.249]	$4.6x10^{-80}$ [$1.5x10^{-83}$, $6.0x10^{-76}$]	10000
	K562	0.177	0.144 [0.143, 0.146]	1.228 [1.214, 1.238]	$2.0x10^{-76}$ [$2.1x10^{-80}$, $6.0x10^{-72}$]	10000
	Nhek	0.179	0.145 [0.143, 0.147]	1.232 [1.218, 1.246]	$1.0x10^{-77}$ [$1.6x10^{-81}$, $2.0x10^{-73}$]	10000
	Nhlf	0.176	0.144 [0.143, 0.146]	1.222 [1.208, 1.236]	$1.2x10^{-76}$ [$1.9x10^{-80}$, $3.3x10^{-72}$]	10000
