# Supplementary material for: Increased ultra-rare variant load in an isolated Scottish population impacts exonic and regulatory regions
Source: PLoS Genet. 2019 Nov 25;15(11):e1008480. doi: 10.1371/journal.pgen.1008480 (PMC6901239; doi:10.1371/journal.pgen.1008480)
Supplement: S6 Table — To annotate the number of variants in a state/cell type class as significantly different, we required at least 95% of the 10,000 subsets to have p-value ≤ 2x10^-4^ (Bonferroni corrected) and no overlap between the 95% CI for the LBC and VIKING median values; there is no significant difference between the two cohorts for known SNPs in any of the considered states/cell types. (PDF) [file pgen.1008480.s019.pdf]

S6 Table. VIKING vs LBC: known SNP load comparison in different chromatin states (alleles per individual per 1Mb).

State	Cell Type	VIKING median	LBC 10k subsets median & 95%CI	VIKING/LBC ratio median & 95%CI	Wilcoxon rank sum test	
					p : median & 95% CI	number of tests with $p \leq 2x10^{-4}$
Promoter	Gm12878	1116	1116 [1115, 1117]	1.000 [0.999, 1.001]	5.9x10^{-1} [9.1x10^{-2}, 9.8x10^{-1}]	0
	H1hesc	1148	1147 [1146, 1147]	1.001 [1.001, 1.002]	8.1x10^{-3} [1.1x10^{-4}, 1.5x10^{-1}]	421
	Hepg2	1205	1204 [1203, 1205]	1.001 [1.000, 1.001]	9.4x10^{-2} [3.1x10^{-3}, 6.9x10^{-1}]	9
	Hmec	1109	1106 [1105, 1108]	1.002 [1.001, 1.003]	1.6x10^{-4} [5.8x10^{-7}, 1.0x10^{-2}]	5327
	Hsmm	1131	1128 [1127, 1129]	1.002 [1.001, 1.003]	4.3x10^{-5} [1.1x10^{-7}, 4.5x10^{-3}]	7205
	Huvec	1127	1125 [1124, 1126]	1.002 [1.001, 1.003]	6.8x10^{-4} [5.0x10^{-6}, 2.6x10^{-2}]	3011
	K562	1133	1131 [1130, 1132]	1.002 [1.001, 1.003]	1.0x10^{-2} [1.3x10^{-4}, 1.9x10^{-1}]	376
	Nhek	1167	1166 [1165, 1167]	1.001 [1.000, 1.002]	1.5x10^{-2} [2.2x10^{-4}, 2.3x10^{-1}]	227
	Nhlf	1143	1141 [1140, 1142]	1.001 [1.000, 1.002]	7.7x10^{-3} [9.2x10^{-5}, 1.7x10^{-1}]	477
Enhancer	Gm12878	1253	1252 [1251, 1253]	1.001 [1.000, 1.002]	1.9x10^{-1} [1.2x10^{-2}, 8.3x10^{-1}]	0
	H1hesc	1403	1403 [1402, 1403]	1.000 [1.000, 1.001]	5.6x10^{-1} [8.1x10^{-2}, 9.8x10^{-1}]	0
	Hepg2	1275	1274 [1273, 1275]	1.001 [1.000, 1.001]	1.6x10^{-1} [7.1x10^{-3}, 8.5x10^{-1}]	0
	Hmec	1365	1364 [1363, 1365]	1.001 [1.000, 1.001]	1.2x10^{-1} [6.1x10^{-3}, 7.3x10^{-1}]	1
	Hsmm	1395	1393 [1393, 1394]	1.001 [1.000, 1.001]	6.5x10^{-2} [2.7x10^{-3}, 5.1x10^{-1}]	10
	Huvec	1329	1327 [1327, 1328]	1.001 [1.000, 1.002]	1.2x10^{-2} [2.0x10^{-4}, 2.0x10^{-1}]	261
	K562	1202	1202 [1201, 1203]	1.000 [0.999, 1.001]	6.4x10^{-1} [1.2x10^{-1}, 9.8x10^{-1}]	0
	Nhek	1353	1351 [1350, 1352]	1.001 [1.001, 1.002]	1.6x10^{-2} [2.7x10^{-4}, 2.4x10^{-1}]	202
	Nhlf	1341	1340 [1339, 1341]	1.001 [1.000, 1.001]	6.6x10^{-2} [2.6x10^{-3}, 5.3x10^{-1}]	13
Insulator	Gm12878	1455	1455 [1454, 1456]	1.000 [0.999, 1.001]	6.7x10^{-1} [1.8x10^{-1}, 9.8x10^{-1}]	0
	H1hesc	1567	1566 [1565, 1567]	1.001 [1.000, 1.001]	4.9x10^{-1} [6.0x10^{-2}, 9.7x10^{-1}]	0
	Hepg2	1504	1505 [1504, 1506]	0.999 [0.998, 1.000]	1.5x10^{-1} [9.0x10^{-3}, 7.8x10^{-1}]	0
	Hmec	1445	1445 [1444, 1447]	1.000 [0.999, 1.000]	5.0x10^{-1} [7.0x10^{-2}, 9.7x10^{-1}]	0
	Hsmm	1494	1494 [1493, 1496]	1.000 [0.999, 1.001]	5.0x10^{-1} [6.3x10^{-2}, 9.8x10^{-1}]	0
	Huvec	1481	1481 [1480, 1482]	1.000 [0.999, 1.001]	6.7x10^{-1} [1.6x10^{-1}, 9.9x10^{-1}]	0
	K562	1497	1497 [1495, 1498]	1.000 [0.999, 1.001]	6.7x10^{-1} [1.6x10^{-1}, 9.8x10^{-1}]	0
	Nhek	1452	1452 [1451, 1453]	1.000 [0.999, 1.001]	6.4x10^{-1} [1.3x10^{-1}, 9.8x10^{-1}]	0
	Nhlf	1427	1428 [1427, 1429]	1.000 [0.999, 1.000]	5.7x10^{-1} [8.7x10^{-2}, 9.8x10^{-1}]	0
Transcription	Gm12878	1023	1022 [1022, 1023]	1.001 [1.000, 1.002]	1.0x10^{-1} [3.6x10^{-3}, 7.0x10^{-1}]	2
	H1hesc	1143	1142 [1141, 1142]	1.001 [1.000, 1.001]	6.9x10^{-3} [6.7x10^{-5}, 1.5x10^{-1}]	599
	Hepg2	1038	1038 [1037, 1039]	1.000 [0.999, 1.001]	2.8x10^{-1} [1.7x10^{-2}, 9.3x10^{-1}]	1
	Hmec	1083	1082 [1082, 1083]	1.001 [1.000, 1.002]	2.8x10^{-2} [5.6x10^{-4}, 3.2x10^{-1}]	79
	Hsmm	1102	1101 [1101, 1102]	1.001 [1.000, 1.001]	1.6x10^{-2} [2.7x10^{-4}, 2.4x10^{-1}]	172
	Huvec	1048	1047 [1046, 1048]	1.001 [1.000, 1.002]	6.3x10^{-2} [1.7x10^{-3}, 5.5x10^{-1}]	21
	K562	1019	1018 [1017, 1019]	1.001 [1.000, 1.001]	1.4x10^{-1} [5.2x10^{-3}, 8.1x10^{-1}]	3
	Nhek	1063	1062 [1061, 1063]	1.001 [1.000, 1.001]	5.0x10^{-2} [1.3x10^{-3}, 4.7x10^{-1}]	33
	Nhlf	1069	1068 [1067, 1069]	1.001 [1.000, 1.002]	4.6x10^{-3} [4.3x10^{-5}, 1.1x10^{-1}]	798
Repressed	Gm12878	1392	1391 [1390, 1392]	1.001 [1.000, 1.002]	1.8x10^{-1} [1.0x10^{-2}, 8.6x10^{-1}]	1
	H1hesc	1220	1219 [1218, 1220]	1.001 [1.000, 1.002]	1.8x10^{-1} [1.2x10^{-2}, 8.3x10^{-1}]	1
	Hepg2	1503	1503 [1502, 1505]	1.000 [0.999, 1.001]	6.8x10^{-1} [1.7x10^{-1}, 9.9x10^{-1}]	0
	Hmec	1438	1438 [1437, 1440]	1.000 [0.999, 1.001]	5.1x10^{-1} [6.5x10^{-2}, 9.8x10^{-1}]	0
	Hsmm	1418	1418 [1417, 1419]	1.000 [0.999, 1.001]	6.6x10^{-1} [1.4x10^{-1}, 9.8x10^{-1}]	0
	Huvec	1367	1368 [1367, 1369]	1.000 [0.999, 1.000]	5.0x10^{-1} [6.4x10^{-2}, 9.7x10^{-1}]	0
	K562	1473	1473 [1472, 1474]	1.000 [0.999, 1.000]	6.7x10^{-1} [1.6x10^{-1}, 9.8x10^{-1}]	0
	Nhek	1524	1524 [1523, 1526]	0.999 [0.999, 1.001]	3.7x10^{-1} [3.6x10^{-2}, 9.6x10^{-1}]	0
	Nhlf	1404	1404 [1402, 1405]	1.000 [0.999, 1.001]	6.6x10^{-1} [1.5x10^{-1}, 9.8x10^{-1}]	0
Heterochromatin	Gm12878	1392	1391 [1390, 1391]	1.001 [1.000, 1.001]	8.9x10^{-4} [6.2x10^{-6}, 3.3x10^{-2}]	2574
	H1hesc	1370	1369 [1369, 1370]	1.001 [1.000, 1.001]	2.5x10^{-3} [2.3x10^{-5}, 6.5x10^{-2}]	1213
	Hepg2	1387	1385 [1385, 1386]	1.001 [1.000, 1.001]	2.3x10^{-4} [1.0x10^{-6}, 1.2x10^{-2}]	4770
	Hmec	1375	1374 [1373, 1374]	1.001 [1.000, 1.001]	1.1x10^{-3} [8.2x10^{-6}, 3.6x10^{-2}]	2295
	Hsmm	1385	1384 [1384, 1385]	1.001 [1.000, 1.001]	3.8x10^{-3} [4.6x10^{-5}, 9.0x10^{-2}]	845
	Huvec	1382	1381 [1380, 1381]	1.001 [1.000, 1.001]	3.8x10^{-4} [1.8x10^{-6}, 1.8x10^{-2}]	3909
	K562	1391	1390 [1389, 1390]	1.001 [1.001, 1.001]	1.1x10^{-4} [4.3x10^{-7}, 7.7x10^{-3}]	6007
	Nhek	1370	1369 [1369, 1370]	1.001 [1.000, 1.001]	4.3x10^{-4} [2.4x10^{-6}, 1.8x10^{-2}]	3725
	Nhlf	1379	1378 [1377, 1378]	1.001 [1.000, 1.001]	2.7x10^{-3} [2.9x10^{-5}, 7.0x10^{-2}]	1168
