# Supplementary material for: Increased ultra-rare variant load in an isolated Scottish population impacts exonic and regulatory regions
Source: PLoS Genet. 2019 Nov 25;15(11):e1008480. doi: 10.1371/journal.pgen.1008480 (PMC6901239; doi:10.1371/journal.pgen.1008480)
Supplement: S7 Table — To annotate the number of variants in a state/cell type class as significantly different, we required at least 95% of the 10,000 subsets to have p-value ≤ 2x10^-4^ (Bonferroni corrected) and no overlap between the 95% CI for the LBC and VIKING median values; non-significant differences shown in grey. (PDF) [file pgen.1008480.s020.pdf]

**S7 Table. VIKING vs LBC: known INDEL load comparison in different chromatin states (alleles per individual per 1Mb).**

| State | Cell Type | VIKING median | LBC 10k subsets median & 95%CI | VIKING/LBC ratio median & 95%CI | Wilcoxon rank sum test: $p$ : median & 95% CI | Wilcoxon rank sum test: number of tests with $p \le 2\times10^{-4}$ |
|---|---|---|---|---|---|---|
| Promoter | Gm12878 | 131.87 | 130.77 [130.57, 130.97] | 1.008 [1.007, 1.010] | $3.8\times10^{-17}$ [$1.9\times10^{-21}$, $3.0\times10^{-13}$] | 10000 |
| | H1hesc | 134.92 | 133.53 [133.33, 133.72] | 1.010 [1.009, 1.012] | $1.3\times10^{-25}$ [$1.1\times10^{-30}$, $6.0\times10^{-21}$] | 10000 |
| | Hepg2 | 130.44 | 129.17 [129.00, 129.37] | 1.010 [1.008, 1.011] | $5.4\times10^{-22}$ [$9.0\times10^{-27}$, $1.4\times10^{-17}$] | 10000 |
| | Hmec | 138.03 | 136.22 [135.98, 136.43] | 1.013 [1.012, 1.015] | $7.1\times10^{-30}$ [$5.0\times10^{-35}$, $4.6\times10^{-25}$] | 10000 |
| | Hsmm | 135.56 | 133.76 [133.57, 133.96] | 1.013 [1.012, 1.015] | $2.3\times10^{-30}$ [$2.7\times10^{-35}$, $1.3\times10^{-25}$] | 10000 |
| | Huvec | 140.53 | 138.65 [138.45, 138.88] | 1.014 [1.012, 1.015] | $1.3\times10^{-28}$ [$1.5\times10^{-33}$, $7.4\times10^{-24}$] | 10000 |
| | K562 | 135.28 | 134.10 [133.93, 134.26] | 1.009 [1.008, 1.010] | $1.3\times10^{-16}$ [$1.2\times10^{-20}$, $6.5\times10^{-13}$] | 10000 |
| | Nhek | 139.76 | 138.24 [138.02, 138.43] | 1.011 [1.010, 1.013] | $7.3\times10^{-26}$ [$7.2\times10^{-31}$, $2.5\times10^{-21}$] | 10000 |
| | Nhlf | 138.09 | 136.52 [136.31, 136.69] | 1.011 [1.010, 1.013] | $6.8\times10^{-29}$ [$9.1\times10^{-34}$, $3.2\times10^{-24}$] | 10000 |
| Enhancer | Gm12878 | 120.80 | 120.01 [119.90, 120.14] | 1.007 [1.005, 1.007] | $9.4\times10^{-19}$ [$5.4\times10^{-23}$, $8.5\times10^{-15}$] | 10000 |
| | H1hesc | 125.50 | 124.70 [124.57, 124.85] | 1.006 [1.005, 1.008] | $2.4\times10^{-21}$ [$4.5\times10^{-26}$, $4.7\times10^{-17}$] | 10000 |
| | Hepg2 | 116.88 | 116.11 [116.00, 116.24] | 1.007 [1.005, 1.008] | $9.7\times10^{-18}$ [$3.9\times10^{-22}$, $1.1\times10^{-13}$] | 10000 |
| | Hmec | 122.97 | 122.27 [122.12, 122.38] | 1.006 [1.005, 1.007] | $1.4\times10^{-23}$ [$2.4\times10^{-28}$, $2.5\times10^{-19}$] | 10000 |
| | Hsmm | 126.19 | 125.34 [125.21, 125.41] | 1.007 [1.006, 1.008] | $7.7\times10^{-25}$ [$3.1\times10^{-29}$, $1.0\times10^{-20}$] | 10000 |
| | Huvec | 128.53 | 127.73 [127.62, 127.88] | 1.006 [1.005, 1.007] | $2.0\times10^{-20}$ [$3.6\times10^{-25}$, $2.9\times10^{-16}$] | 10000 |
| | K562 | 112.07 | 111.42 [111.29, 111.53] | 1.006 [1.005, 1.007] | $2.1\times10^{-17}$ [$6.5\times10^{-22}$, $2.1\times10^{-13}$] | 10000 |
| | Nhek | 124.47 | 123.64 [123.51, 123.77] | 1.007 [1.006, 1.008] | $4.3\times10^{-24}$ [$5.6\times10^{-29}$, $1.1\times10^{-19}$] | 10000 |
| | Nhlf | 126.22 | 125.39 [125.28, 125.52] | 1.007 [1.006, 1.008] | $9.1\times10^{-24}$ [$1.5\times10^{-28}$, $2.6\times10^{-19}$] | 10000 |
| Insulator | Gm12878 | 138.49 | 137.68 [137.42, 137.95] | 1.006 [1.004, 1.008] | $9.1\times10^{-4}$ [$7.3\times10^{-6}$, $3.0\times10^{-2}$] | 2454 |
| | H1hesc | 136.59 | 135.99 [135.76, 136.22] | 1.004 [1.003, 1.006] | $7.6\times10^{-5}$ [$2.6\times10^{-7}$, $5.7\times10^{-3}$] | 6533 |
| | Hepg2 | 149.21 | 148.61 [148.26, 148.95] | 1.004 [1.002, 1.006] | $4.5\times10^{-3}$ [$5.7\times10^{-5}$, $9.2\times10^{-2}$] | 741 |
| | Hmec | 145.93 | 145.45 [145.05, 145.77] | 1.003 [1.001, 1.006] | $6.0\times10^{-3}$ [$7.5\times10^{-5}$, $1.2\times10^{-1}$] | 579 |
| | Hsmm | 146.92 | 146.79 [146.54, 147.12] | 1.001 [0.999, 1.003] | $3.3\times10^{-1}$ [$3.1\times10^{-2}$, $9.5\times10^{-1}$] | 0 |
| | Huvec | 145.04 | 144.77 [144.44, 145.04] | 1.002 [1.000, 1.004] | $9.7\times10^{-2}$ [$5.1\times10^{-3}$, $5.8\times10^{-1}$] | 2 |
| | K562 | 133.94 | 133.16 [132.90, 133.48] | 1.006 [1.004, 1.008] | $4.4\times10^{-6}$ [$6.8\times10^{-9}$, $6.4\times10^{-4}$] | 9281 |
| | Nhek | 143.94 | 143.37 [143.06, 143.63] | 1.004 [1.002, 1.006] | $1.8\times10^{-4}$ [$9.3\times10^{-7}$, $1.0\times10^{-2}$] | 5209 |
| | Nhlf | 128.74 | 128.17 [127.94, 128.40] | 1.004 [1.003, 1.006] | $1.8\times10^{-5}$ [$5.1\times10^{-8}$, $1.5\times10^{-3}$] | 8390 |
| Transcription | Gm12878 | 118.45 | 117.62 [117.48, 117.71] | 1.007 [1.006, 1.008] | $3.5\times10^{-29}$ [$2.1\times10^{-34}$, $2.3\times10^{-24}$] | 10000 |
| | H1hesc | 123.56 | 122.68 [122.59, 122.80] | 1.007 [1.006, 1.008] | $4.5\times10^{-41}$ [$2.8\times10^{-46}$, $4.8\times10^{-36}$] | 10000 |
| | Hepg2 | 118.89 | 118.07 [117.95, 118.15] | 1.007 [1.006, 1.008] | $1.1\times10^{-33}$ [$5.0\times10^{-39}$, $1.3\times10^{-28}$] | 10000 |
| | Hmec | 121.93 | 121.12 [121.03, 121.23] | 1.007 [1.006, 1.007] | $7.3\times10^{-32}$ [$4.3\times10^{-37}$, $5.9\times10^{-27}$] | 10000 |
| | Hsmm | 122.49 | 121.64 [121.55, 121.74] | 1.007 [1.006, 1.008] | $4.8\times10^{-37}$ [$1.8\times10^{-42}$, $7.0\times10^{-32}$] | 10000 |
| | Huvec | 120.42 | 119.57 [119.46, 119.67] | 1.007 [1.006, 1.008] | $5.3\times10^{-32}$ [$4.2\times10^{-37}$, $4.4\times10^{-27}$] | 10000 |
| | K562 | 118.77 | 117.90 [117.80, 118.02] | 1.007 [1.006, 1.008] | $2.2\times10^{-33}$ [$7.2\times10^{-39}$, $2.4\times10^{-28}$] | 10000 |
| | Nhek | 120.05 | 119.18 [119.10, 119.29] | 1.007 [1.006, 1.008] | $1.2\times10^{-34}$ [$5.6\times10^{-40}$, $1.7\times10^{-29}$] | 10000 |
| | Nhlf | 121.62 | 120.76 [120.64, 120.84] | 1.007 [1.006, 1.008] | $2.9\times10^{-34}$ [$2.1\times10^{-39}$, $2.8\times10^{-29}$] | 10000 |
| Repressed | Gm12878 | 123.70 | 122.97 [122.83, 123.11] | 1.006 [1.005, 1.007] | $7.0\times10^{-13}$ [$9.5\times10^{-17}$, $1.7\times10^{-9}$] | 10000 |
| | H1hesc | 117.27 | 116.31 [116.13, 116.58] | 1.008 [1.006, 1.010] | $9.9\times10^{-10}$ [$4.5\times10^{-13}$, $8.7\times10^{-7}$] | 10000 |
| | Hepg2 | 128.09 | 127.42 [127.25, 127.58] | 1.005 [1.004, 1.007] | $3.6\times10^{-9}$ [$1.9\times10^{-12}$, $2.5\times10^{-6}$] | 9998 |
| | Hmec | 122.80 | 122.19 [122.00, 122.37] | 1.005 [1.003, 1.006] | $2.3\times10^{-6}$ [$3.2\times10^{-9}$, $4.4\times10^{-4}$] | 9495 |
| | Hsmm | 126.57 | 126.11 [125.97, 126.26] | 1.004 [1.002, 1.005] | $1.6\times10^{-7}$ [$1.3\times10^{-10}$, $5.0\times10^{-5}$] | 9936 |
| | Huvec | 125.25 | 124.82 [124.67, 124.95] | 1.003 [1.002, 1.005] | $2.5\times10^{-6}$ [$5.7\times10^{-9}$, $3.6\times10^{-4}$] | 9547 |
| | K562 | 127.10 | 126.46 [126.34, 126.60] | 1.005 [1.004, 1.006] | $8.4\times10^{-15}$ [$4.2\times10^{-19}$, $4.7\times10^{-11}$] | 10000 |
| | Nhek | 128.13 | 127.45 [127.33, 127.60] | 1.005 [1.004, 1.006] | $2.5\times10^{-11}$ [$6.1\times10^{-15}$, $4.1\times10^{-8}$] | 10000 |
| | Nhlf | 120.58 | 119.88 [119.78, 120.06] | 1.006 [1.004, 1.007] | $1.5\times10^{-12}$ [$4.4\times10^{-16}$, $2.1\times10^{-9}$] | 10000 |
| Heterochromatin | Gm12878 | 137.82 | 137.11 [137.05, 137.17] | 1.005 [1.005, 1.006] | $1.2\times10^{-42}$ [$9.3\times10^{-48}$, $1.3\times10^{-37}$] | 10000 |
| | H1hesc | 136.72 | 136.10 [136.03, 136.15] | 1.005 [1.004, 1.005] | $1.2\times10^{-39}$ [$8.0\times10^{-45}$, $1.5\times10^{-24}$] | 10000 |
| | Hepg2 | 138.21 | 137.50 [137.43, 137.57] | 1.005 [1.005, 1.006] | $3.8\times10^{-42}$ [$2.7\times10^{-47}$, $3.5\times10^{-37}$] | 10000 |
| | Hmec | 137.03 | 136.35 [136.28, 136.42] | 1.005 [1.005, 1.006] | $7.6\times10^{-43}$ [$5.3\times10^{-48}$, $1.1\times10^{-37}$] | 10000 |
| | Hsmm | 137.74 | 137.08 [136.99, 137.15] | 1.005 [1.004, 1.005] | $1.0\times10^{-39}$ [$9.4\times10^{-45}$, $8.7\times10^{-35}$] | 10000 |
| | Huvec | 137.09 | 136.39 [136.32, 136.46] | 1.005 [1.005, 1.006] | $3.7\times10^{-44}$ [$3.3\times10^{-49}$, $3.6\times10^{-39}$] | 10000 |
| | K562 | 139.31 | 138.58 [138.53, 138.66] | 1.005 [1.005, 1.006] | $2.1\times10^{-41}$ [$2.8\times10^{-46}$, $1.4\times10^{-36}$] | 10000 |
| | Nhek | 137.52 | 136.85 [136.77, 136.91] | 1.005 [1.004, 1.006] | $3.6\times10^{-41}$ [$2.9\times10^{-46}$, $4.5\times10^{-36}$] | 10000 |
| | Nhlf | 138.56 | 137.41 [137.32, 137.46] | 1.005 [1.004, 1.005] | $1.6\times10^{-40}$ [$1.2\times10^{-45}$, $1.8\times10^{-35}$] | 10000 |
