# Supplementary material for: Increased ultra-rare variant load in an isolated Scottish population impacts exonic and regulatory regions
Source: PLoS Genet. 2019 Nov 25;15(11):e1008480. doi: 10.1371/journal.pgen.1008480 (PMC6901239; doi:10.1371/journal.pgen.1008480)
Supplement: S8 Table — To annotate a difference as significantly different, we required at least 95% of the 10,000 subsets to have p-value ≤ 0.0125 (Bonferroni corrected) and no overlap between the 95% CI for the LBC and VIKING median values. The ROHs used for the analysis are filtered to exclude ROH regions with poor SNP density (see S8 Fig). (PDF) [file pgen.1008480.s021.pdf]

S8 Table. Comparison of the ROH regions discovered in VIKING and LBC.

		VIKING	LBC (10k sub-samples)			VIKING/LBC ratio			Wilcoxon rank sum test			
		median	median	95% LO	95% HI	median	95% LO	95% HI	median	95% LO	95% HI	number of tests with $p \leq 0.0125$
Intermediate	number	135	142	140	143	0.951	0.944	0.964	9.4×10^{-12}	3.3×10^{-15}	1.0×10^{-8}	10000
	length	97.1 Mb	102.4 Mb	101.2 Mb	103.9Mb	0.948	0.935	0.959	8.5×10^{-12}	2.5×10^{-15}	1.0×10^{-8}	10000
Long	number	3	1	1	2	3.000	1.500	3.000	2.7×10^{-22}	3.8×10^{-26}	1.1×10^{-18}	10000
	length	9.6 Mb	4.2 Mb	3.3 Mb	4.5 Mb	2.307	2.155	2.934	1.7×10^{-31}	3.1×10^{-35}	7.3×10^{-28}	10000
