# Supplementary material for: Increased ultra-rare variant load in an isolated Scottish population impacts exonic and regulatory regions
Source: PLoS Genet. 2019 Nov 25;15(11):e1008480. doi: 10.1371/journal.pgen.1008480 (PMC6901239; doi:10.1371/journal.pgen.1008480)
Supplement: S9 Table — From the gnomAD dataset we report the MAF for the population with the maximum MAF for the variant; gnomADg is WGS data (n = 15,496) and gnomADe is WES data (v2.1.1, n = 125,748). The p-value for the VIKING vs gnomADg enrichment for a variant is calculated using Fisher’s Exact Test. (PDF) [file pgen.1008480.s022.pdf]

S9 Table. The 13 exonic variants found to be significantly enriched in VIKING compared to gnomADg (Fisher's Exact Test) in genes predicted to be largely intolerant to variation and for which a strong evidence of gene-trait association ($p \leq 5x10^{-8}$) is reported in the GWAS Catalog (v1.0.1).

chr	pos	id	ref	alt	VIKING MAF	LBC MAF	gnomADg MAF (max)	gnomADe MAF (max)	Gene	Effect	VIKING/gnomADg enrichment p-value	gene-trait correlation (p-value ≤ $5x10^{-8}$ in GWAS Catalog v1.0.1)
19	49,232,226	rs2287922	G	A	0.625	0.565	0.4815	0.4918	RASIP1	missense_variant	$4.0x10^{-19}$	Retinal vascular calibre, Urinary metabolites (H-NMR features), Inflammatory skin disease, C-reactive protein levels or LDL-cholesterol levels (pleiotropy), Mean platelet volume
17	48,649,340	rs768796872	G	A	0.013	0	0	0.0001651	CACNA1G	missense_variant	$2.3x10^{-16}$	Alzheimer's disease (cognitive decline)
5	98,215,264	rs370760984	C	T	0.011	0.0008651	0.0003332	0.0002320	CHD1	missense_variant	$7.3x10^{-16}$	Eosinophil percentage of white cells, Eosinophil counts, Eosinophil percentage of granulocytes, Neutrophil percentage of granulocytes
3	10,400,565	rs774434270	C	T	0.005576	0.0004325	0	0.00003266	ATP2B2	missense_variant	$4.0x10^{-13}$	Autism spectrum disorder or schizophrenia
19	42,753,837	rs368169058	G	A	0.013	0	0	0.0002846	ERF	missense_variant	$7.6x10^{-13}$	Monocyte count
14	79,423,644	rs139593796	G	A	0.011	0	0.0001332	0.0001055	NRXN3	missense_variant	$3.7x10^{-12}$	Waist circumference, Body mass index, Obesity, Waist-hip ratio, Hip circumference, Cerebrospinal fluid biomarker levels, Initial pursuit acceleration
20	47,247,325	rs138500849	A	T	0.022	0.002595	0.002045	0.001959	PREX1	missense_variant	$4.2x10^{-11}$	Colorectal cancer, Multiple myeloma, Diastolic blood pressure, Intelligence (multi-trait analysis)
17	48,703,924	rs762245146	C	G	0.011	0	0.00006696	0.0001834	CACNA1G	missense_variant	$1.4x10^{-10}$	Alzheimer's disease (cognitive decline)
22	40,042,737	rs201769752	T	C	0.011	0.001298	0.0002004	0.0008119	CACNA1I	missense_variant	$2.1x10^{-10}$	IgG glycosylation, Schizophrenia, Autism spectrum disorder or schizophrenia, Cognitive ability (multi-trait analysis), Intelligence (multi-trait analysis)
10	78,647,084	.	G	C	0.007435	0.0004325	0	0	KCNMA1	missense_variant	$1.6x10^{-9}$	Obesity, Hypospadias, Myopia
10	78,708,961	rs148156399	A	G	0.007435	0	0	0.0000088	KCNMA1	missense_variant	$8.8x10^{-9}$	Obesity, Hypospadias, Myopia
3	47,162,897	rs114719990	T	C	0.013	0.0008651	0.001399	0.001446	SETD2	missense_variant	$3.4x10^{-7}$	HDL cholesterol levels, Macrophage inflammatory protein 1b levels, Monocyte count, Lymphocyte counts, Lymphocyte percentage of white cells, Monocyte percentage of white cells, Granulocyte percentage of myeloid white cells, White blood cell count, Platelet distribution width
18	52,928,743	rs147445499	G	A	0.011	0.0004325	0.0008661	0.001184	TCF4	missense_variant	$4.7x10^{-7}$	Schizophrenia, Fuchs's corneal dystrophy, Sclerosing cholangitis and ulcerative colitis (combined), Autism spectrum disorder or schizophrenia, Neuroticism
