# Supplementary material for: Increased ultra-rare variant load in an isolated Scottish population impacts exonic and regulatory regions
Source: PLoS Genet. 2019 Nov 25;15(11):e1008480. doi: 10.1371/journal.pgen.1008480 (PMC6901239; doi:10.1371/journal.pgen.1008480)
Supplement: S10 Table — From the gnomAD dataset we report the MAF for the population with the maximum MAF for the variant; gnomADg is WGS data (n = 15,496). The p-value for the VIKING vs gnomADg enrichment for a variant is calculated using Fisher’s Exact Test. (PDF) [file pgen.1008480.s023.pdf]

S10 Table. The 6 rare variants (gnomADg MAF < 0.05, Shetland MAF ≤ 0.1) predicted to be eQTLs (GTEx v7, qval ≤ 0.05) and to affect the expression of 6 distinct genes.

chr	pos	id	ref	alt	VIKING MAF	LBC MAF	gnomADg MAF (max)	Mapped Gene	Tissue	VIKING/gnomADg enrichment p-value	gene-trait correlation (p-value ≤ $5x10^{-8}$ in GWAS Catalog v1.0.1)
1	154,909,169	rs17356361	C	T	0.097	0.043	0.034	PMVK	Esophagus_Mucosa	$2.5x10^{-9}$	Atrial fibrillation, Lung function (FEV1/FVC), Parkinson's disease
11	65,379,532	rs138504384	GTC	G	0.067	0.031	0.021	ZNHIT2	Cells_Transformed_fibroblasts	$6.9x10^{-9}$	
9	136,341,547	rs117965396	C	T	0.061	0.026	0.019	CACFD1	Esophagus_Muscularis<br>Muscle_Skeletal	$1.4x10^{-8}$	
1	155,066,403	rs138207102	C	T	0.052	0.022	0.015	CHRNB2	Brain_Putamen_basal_ganglia	$3.3x10^{-8}$	Smoking behaviour (cigarettes smoked / day)
10	81,839,199	rs150170834	G	A	0.046	0.019	0.013	FAM213A	Liver	$3.4x10^{-8}$	Lung function (FEV1/FVC), Heel bone mineral density, Blood protein levels, Systolic blood pressure, Intraocular pressure, Sunburns
1	46,935,356	rs150548439	A	C	0.063	0.033	0.022	LRRC41	Artery_Aorta	$2.0x10^{-7}$	Menopause (age at onset)
