# Supplementary material for: Increased ultra-rare variant load in an isolated Scottish population impacts exonic and regulatory regions
Source: PLoS Genet. 2019 Nov 25;15(11):e1008480. doi: 10.1371/journal.pgen.1008480 (PMC6901239; doi:10.1371/journal.pgen.1008480)
Supplement: S11 Table — The analysis is based on high-quality SNPs/INDELs discovered in the callable regions of the 22 autosomal chromosomes in the two cohorts of unrelated individuals, split to known variants (present in gnomADg at any frequency) and ultra-rare variants (not found in any gnomADg population). All sites with missing genotype(s) were excluded. The means and standard deviations for each frequency were computed based on subsampling the two cohorts to 50 individuals each repeated 100 times (also see S6 Fig). (PDF) [file pgen.1008480.s024.pdf]

S11 Table. Mean and standard deviation of proportion of sites with particular number of MAF alleles in the VIKING and LBC cohorts.

		number MAF alleles in known SNPs							number MAF alleles in ultra-rare SNPs		
		**1**	**2**	**3**	**4-5**	**6-10**	**11-20**	**21-50**	**1**	**2**	**3+**
**VIK**	mean	0.190	0.090	0.060	0.077	0.120	0.154	0.320	0.881	0.093	0.027
	s.d.	$5.6x10^{-17}$	$8.4x10^{-17}$	$2.0x10^{-3}$	$4.5x10^{-3}$	$2.0x10^{-16}$	$4.9x10^{-3}$	$2.2x10^{-16}$	$6.8x10^{-3}$	$5.6x10^{-3}$	$4.6x10^{-3}$
**LBC**	mean	0.220	0.080	0.050	0.070	0.115	0.150	0.310	0.980	0.010	0.010
	s.d.	$1.1x10^{-16}$	$5.6x10^{-17}$	$9.8x10^{-17}$	$8.4x10^{-17}$	$5.0x10^{-3}$	$2.5x10^{-16}$	$5.6x10^{-16}$	$4.5x10^{-16}$	$7.0x10^{-18}$	$7.0x10^{-18}$

		number MAF alleles in known INDELs							number MAF alleles in ultra-rare INDELs		
		**1**	**2**	**3**	**4-5**	**6-10**	**11-20**	**21-50**	**1**	**2**	**3+**
**VIK**	mean	0.186	0.089	0.057	0.079	0.120	0.160	0.320	0.863	0.096	0.040
	s.d.	$4.9x10^{-3}$	$3.0x10^{-3}$	$4.6x10^{-3}$	$2.6x10^{-3}$	$2.0x10^{-16}$	$1.1x10^{-16}$	$2.2x10^{-16}$	$7.1x10^{-3}$	$6.0x10^{-3}$	$1.4x10^{-3}$
**LBC**	mean	0.211	0.080	0.050	0.070	0.120	0.150	0.310	0.970	0.010	0.020
	s.d.	$2.9x10^{-3}$	$5.6x10^{-17}$	$9.8x10^{-17}$	$8.4x10^{-17}$	$2.0x10^{-16}$	$2.5x10^{-16}$	$5.6x10^{-16}$	$7.8x10^{-16}$	$1.7x10^{-3}$	$1.4x10^{-17}$
