# Supplementary material for: Increased ultra-rare variant load in an isolated Scottish population impacts exonic and regulatory regions
Source: PLoS Genet. 2019 Nov 25;15(11):e1008480. doi: 10.1371/journal.pgen.1008480 (PMC6901239; doi:10.1371/journal.pgen.1008480)
Supplement: S12 Table — Applied filtering criteria are denoted with ‘yes’. (PDF) [file pgen.1008480.s025.pdf]

S12 Table. Functional VIKING variants enriched in genes largely intolerant to variation.

	total number enriched variants	filtering criteria			number filtered enriched variants
		CADD >= 20	pLI >= 0.8	missense z-score >= 3	
**SNPs**					
stop_gained	557		yes		23
splice_acceptor_variant	9		yes		1
splice_donor_variant	27		yes		3
start_lost	46		yes		5
stop_lost	23		yes		2
missense_variant	26054	yes		yes	1165
**INDELs**					
frameshift_variant	749		yes		50
inframe_insertion	133	yes	yes	yes	0
inframe_deletion	396	yes	yes	yes	12
